# Neoadjuvant Regimens and Their Impact on Adjuvant T-DM1 Outcomes in HER2-Positive Early Breast Cancer

**DOI:** 10.3390/medicina61111966

**Published:** 2025-11-01

**Authors:** Ahmet Burak Agaoglu, Atike Pinar Erdogan, Ferhat Ekinci, Mustafa Sahbazlar, Guler Nur Tekustun, Ozgur Tanriverdi, Salih Tunbekici, Erdem Goker, Mehmet Sinan Akarca, Can Cangur, Taliha Guclu Kantar, Sedat Biter, Ertugrul Bayram, Gokhan Colak, Bilgin Demir, Hasan Basir, Vehbi Ercolak

**Affiliations:** 1 Department of Medical Oncology, Faculty of Medicine, Manisa Celal Bayar University, Manisa 45030, Turkey; dr_pinarcan@yahoo.com (A.P.E.); drferhatekinci@hotmail.com (F.E.); m_sahbazlar@hotmail.com (M.S.); 2 Department of Medical Oncology, Faculty of Medicine, Mugla Sitki Kocman University, Mugla 48000, Turkey; gulernurceltik@gmail.com (G.N.T.); dr.ozgur.tanriverdi@gmail.com (O.T.); 3 Department of Medical Oncology, Faculty of Medicine, Ege University, Izmir 35100, Turkey; slhtnbkc@yahoo.com (S.T.); erdem.goker@ege.edu.tr (E.G.); 4 Department of Medical Oncology, Faculty of Medicine, Dokuz Eylul University, Izmir 35340, Turkey; akarcasinan@gmail.com; 5 Department of Medical Oncology, Faculty of Medicine, Selcuk University, Konya 42075, Turkey; ccangur@yahoo.com; 6 Department of Medical Oncology, Faculty of Medicine, Pamukkale University, Denizli 20070, Turkey; talihaguclu@hotmail.com; 7 Department of Medical Oncology, Faculty of Medicine, Cukurova University, Adana 01330, Turkey; sedatb23@hotmail.com (S.B.); ertugrulbayram84@gmail.com (E.B.); 8 Department of Medical Oncology, Faculty of Medicine, Aydin Adnan Menderes University, Aydin 09100, Turkey; drgokhancolak@gmail.com (G.C.); bilgin287@hotmail.com (B.D.); 9 Department of Medical Oncology, Faculty of Medicine, Mersin University, Mersin 33110, Turkey; drhasanbasir@gmail.com (H.B.); vehbiercolak@mersin.edu.tr (V.E.)

**Keywords:** breast neoplasms, HER2-positive, post-neoadjuvant treatment outcomes, real-world outcomes

## Abstract

*Background and Objectives*: In early-stage HER2-positive breast cancer, ado-trastuzumab emtansine (T-DM1) has been adopted as the preferred adjuvant approach for patients left with residual invasive disease despite neoadjuvant therapy. The influence of different neoadjuvant regimens on subsequent outcomes in real-world settings remains uncertain. *Materials and Methods*: From 2019 to 2025, 102 patients treated with adjuvant T-DM1 following surgery after neoadjuvant chemotherapy were retrospectively assessed. Neoadjuvant regimens included doxorubicin plus cyclophosphamide followed by trastuzumab-paclitaxel, doxorubicin plus cyclophosphamide with pertuzumab–trastuzumab–docetaxel, or docetaxel–carboplatin–trastuzumab–pertuzumab. Clinical features, treatment response, survival, and toxicity were evaluated. *Results*: The mean age of the cohort was 49.7 years, and the majority of patients (80.4%) were aged 40 years or older. Hormone receptor positivity was 82.0%, and invasive ductal carcinoma accounted for 97.1% of cases. Regional responses included 39.2% with axillary pCR despite residual breast lesions, and 5.9% with breast pCR accompanied by axillary disease. Kaplan–Meier analysis demonstrated disease-free survival rates of 100%, 95.2%, and 92.2% at 1, 3, and 5 years, respectively. Adverse events were predominantly grade 1–2, while grade 3–4 toxicities occurred in under 5% of the cohort. Baseline characteristics varied across regimens, reflecting real-world treatment preferences, but survival outcomes remained comparable. *Conclusions*: Adjuvant T-DM1 was associated with high survival rates and manageable toxicity across different neoadjuvant regimens, underscoring its consistent benefit in routine clinical practice.

## 1. Introduction

Globally, breast cancer remains the predominant malignancy affecting women and is still responsible for a substantial proportion of cancer mortality [1]. Between 15% and 20% of breast cancers are characterized by amplification or overexpression of HER2, historically regarded as an aggressive subtype with inferior survival outcomes [2]. Introducing trastuzumab as part of adjuvant therapy substantially changed the prognosis of HER2-positive breast cancer [3], and subsequent studies demonstrated that shorter treatment durations can provide non-inferior efficacy compared with the standard 12-month regimen [4].

The advent of dual HER2 inhibition using pertuzumab and trastuzumab has led to greater therapeutic efficacy in both preoperative and postoperative treatment contexts. The addition of pertuzumab increased pathological complete response (pCR) rates in the neoadjuvant context and prolonged invasive disease-free survival when combined with trastuzumab in the adjuvant setting [5,6]. Nonetheless, even with these optimized HER2-directed regimens, patients harboring residual invasive disease after neoadjuvant treatment continue to experience a notable risk of recurrence [7].

The pivotal phase III KATHERINE trial demonstrated that switching adjuvant therapy from trastuzumab to ado-trastuzumab emtansine (T-DM1) significantly improved invasive disease-free survival among individuals harboring residual invasive disease following neoadjuvant treatment [8]. Subsequent analyses confirmed consistent benefit across clinically relevant subgroups [9], and additional data from the ATEMPT trial highlighted positive long-term findings for T-DM1 in stage I HER2-positive patients [10]. Together, these findings resulted in adjuvant T-DM1 being regarded as the frontline strategy for patients with high-risk, early-stage HER2-positive disease.

Nevertheless, important issues remain concerning the impact of different neoadjuvant strategies—such as anthracycline-containing versus anthracycline-free approaches—on subsequent outcomes with adjuvant T-DM1. Evidence from real-world practice addressing this issue is limited and heterogeneous [11,12].

Despite these advances, uncertainty remains regarding how different neoadjuvant regimens influence subsequent outcomes with adjuvant T-DM1. Through the evaluation of clinical characteristics, treatment responses, and survival across different regimens, our study aims to determine whether the choice of neoadjuvant strategy modifies the consistent survival benefit of T-DM1 reported in clinical trials.

## 2. Materials and Methods

Over the period extending from January 2019 to July 2025, clinical data from 102 patients with histopathologically confirmed HER2-positive early-stage breast carcinoma were retrospectively analyzed in this multicenter investigation involving nine oncology centers across Turkey. Each patient proceeded to curative-intent surgery after receiving neoadjuvant chemotherapy and was then managed with adjuvant T-DM1. Inclusion criteria required individuals to be 18 years or older, with invasive breast cancer verified on histology, HER2 positivity defined as IHC 3+ or ISH amplification, and residual invasive disease present within the breast and/or axilla at the time of surgery. Patients who achieved total pathological complete response (ypT0/is ypN0) after neoadjuvant therapy were not eligible, as adjuvant T-DM1 is indicated only for those with residual invasive disease. Patients were not eligible if metastatic disease was present at diagnosis or if their clinical records were incomplete.

Neoadjuvant chemotherapy regimens varied across institutions, reflecting real-world practice. The most frequently used approaches were doxorubicin plus cyclophosphamide followed by trastuzumab and paclitaxel (AC → TH); doxorubicin plus cyclophosphamide followed by pertuzumab, trastuzumab, and docetaxel (AC → THP); and the anthracycline-free combination of docetaxel, carboplatin, trastuzumab, and pertuzumab (TCHP). Completion of the neoadjuvant regimen was followed in all cases by definitive surgical resection aiming at curative intent.

Patients with residual invasive disease subsequently received adjuvant T-DM1 (3.6 mg/kg intravenously, repeated every 3 weeks for up to 14 cycles) according to current treatment guidelines. Dose reductions and schedule modifications were permitted based on toxicity or at the physician’s discretion. Treatment adherence and the number of completed cycles were also recorded. 

Data regarding demographic variables, tumor characteristics, treatment specifications, and pCR status were retrospectively obtained from medical documentation. Pathological response was evaluated separately for the breast and axillary regions, and regional pCR was defined as the absence of residual invasive carcinoma within the respective site. The primary endpoint was disease-free survival (DFS), defined as the time from surgery to the first occurrence of recurrence, progression, or death. Secondary endpoints comprised breast pCR or axillary pCR rates, as well as documentation of adverse events considered related to therapy, which were evaluated and scored based on the standards outlined in the Common Terminology Criteria for Adverse Events, version 5.0. Only adverse events occurring during adjuvant T-DM1 treatment were included in this analysis. Baseline laboratory parameters are provided in the Supplementary Materials (Table S1).

### Statistical Analysis

Data processing and statistical evaluations were performed employing SPSS, release 15.0 (SPSS Inc., Chicago, IL, USA). Descriptive statistics were generated to summarize the dataset in detail. Continuous variables were presented as mean values accompanied by their standard deviations, whereas categorical variables were summarized as absolute numbers together with their corresponding percentages. Group comparisons of categorical variables were evaluated primarily with the chi-square test, and Fisher’s exact test was considered where expected counts were too small to ensure validity. For time-to-event outcomes, survival analyses were undertaken using the Kaplan–Meier method, providing estimates of disease-free survival probabilities over time. Survival curves were compared across subgroups using the log-rank test, allowing assessment of potential differences in event-free survival between treatment categories. In addition, all *p*-values are reported as two-sided, with statistical significance predefined at a threshold of less than 0.05. This analytic framework was selected to ensure reproducibility and consistency with established standards in oncological clinical research.

This study was carried out on 15 January 2025 in accordance with the Declaration of Helsinki, by decision no: E-20478486-050.04-937858, and was granted approval from the Ethics Committee of Manisa Celal Bayar University, Faculty of Medicine. Subsequently, additional approval was granted to extend the study to nine centers (approval date: 9 July 2025; decision no: E-20478486-050.04-1051328).

## 3. Results

### 3.1. Patient Characteristics

Between 2019 and 2025, a total of 102 patients, with a mean age of 49.7 ± 11.0 years (range, 21–73), were included in this analysis, of whom 80.4% were 40 years or older. Among the study population, 77.5% had an ECOG performance status of 0, and 58.8% were postmenopausal. The majority of tumors were cT2 (49.0%) and cN1 (46.1%) at diagnosis. Hormone receptor expression was common, observed in 80.4% of patients for estrogen receptor (ER) and 74.5% for progesterone receptor (PR). HER2 status was IHC 3+ in 61.8% and IHC 2+/FISH+ in 38.2%. Elevated Ki-67 expression (≥14%) was present in 84.3% of the study population. Baseline information regarding the cohort’s clinical and pathological features is illustrated in Table 1.

### 3.2. Treatment Modalities and Pathological Response

Most patients received anthracycline-containing neoadjuvant therapy (76.5%), whereas 23.5% received an anthracycline-free TCHP regimen. The predominant neoadjuvant approach was AC → THP (68.6%), followed by TCHP (22.5%) and AC → TH (8.8%).

Breast-conserving surgery was carried out in 46.1% of cases, whereas 53.9% underwent mastectomy. pCR was obtained in the breast in 6 patients (5.9%) and in the axilla in 40 patients (39.2%). Notably, no recurrence was observed among patients with breast pCR, while only one recurrence occurred in those with axillary pCR. Nearly all patients received adjuvant radiotherapy (93.1%), and endocrine therapy was administered when indicated (79.4%). The mean follow-up duration was 33.9 ± 14.6 months (range, 13–67), during which recurrence or metastasis occurred in 4.9% of patients. Treatment modalities, pathological findings, and outcomes are summarized in Table 2.

Among patients treated with AC → THP, 8 individuals received ≤7 cycles of adjuvant T-DM1, and 2 recurrence events were observed within this subgroup.

### 3.3. Comparison According to Neoadjuvant Regimen

When stratified by neoadjuvant regimen, significant differences were observed in primary tumor stage (T, *p* = 0.001) and lymph node stage (N, *p* = 0.007). Patients in the AC → TH group more frequently presented with cT1 and node-negative disease, whereas those in the AC → THP and TCHP groups more often had more advanced T and N stage at diagnosis. Other baseline variables, including menopausal status, hormone receptor expression, Ki-67, and histological grade, were generally comparable across the three groups (Table 3). Given the limited cohort size in the AC → TH arm, caution is warranted in interpreting subgroup comparisons, since statistical power may be reduced.

Treatment-related characteristics showed no notable differences between the three groups regarding axillary pCR, number of dissected lymph nodes, or receipt of adjuvant T-DM1, radiotherapy, or endocrine therapy. However, breast-conserving surgery was more frequent in the AC → TH group (77.8%) compared with the other regimens (*p* = 0.044), although this finding needs to be interpreted carefully in light of the small number of patients. Recurrence rates did not differ significantly across regimens (Table 4).

### 3.4. Adverse Events

Overall, 75.5% of patients experienced low-grade (1–2) toxicities, whereas higher-grade (3–4) complications were uncommon (3.9%). The most common severe toxicities were thrombocytopenia (4.9%), elevated liver enzymes (4.9%), and anemia (2.9%). Skin toxicity due to radiotherapy was mostly grade 1–2, and hypokalemia was observed only in grade 1–2 cases. The spectrum of toxicities is summarized in Table 5.

The distribution of adverse events differed significantly across neoadjuvant regimens (*p* = 0.018), a finding largely attributable to differences in the incidence of grade 1–2 toxicities. Clinically, however, most toxicities remained mild, and grade 3–4 events were rare across all groups (Table 6).

### 3.5. Survival Outcomes

**Figure 1:**
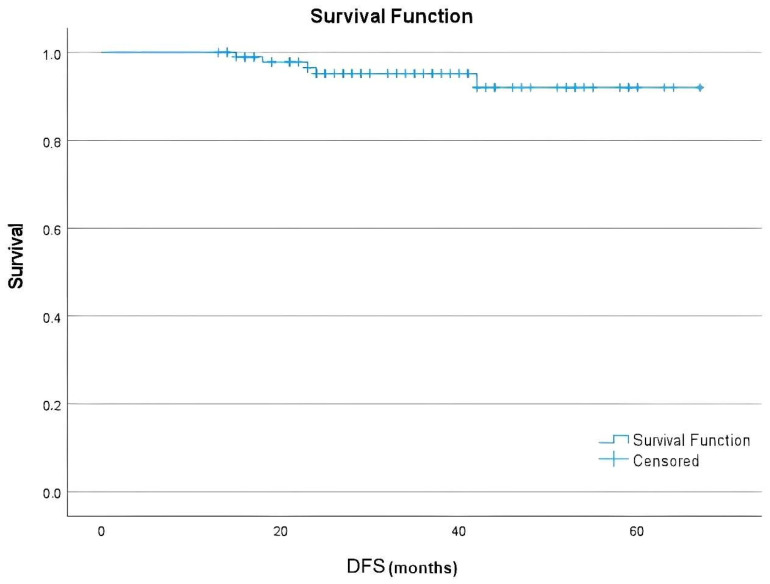
Kaplan–Meier plot depicting disease-free survival in the entire study cohort treated with adjuvant T-DM1.

Kaplan–Meier analysis demonstrated an estimated mean DFS of 64 months (95% CI: 61.4–66.6). The estimated 1-, 3-, and 5-year DFS rates were 100% (95% CI: 100–100), 95.2% (95% CI: 89.6–100.0), and 92.2% (95% CI: 83.0–100.0), respectively (Figure 1). Median DFS was not reached during the follow-up period. Given the very low number of DFS events, the Kaplan–Meier results should be interpreted with caution.

**Figure 2:**
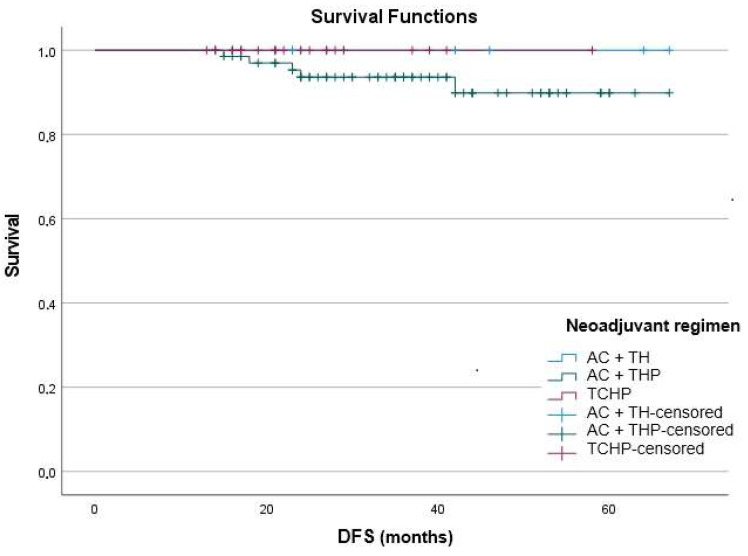
Kaplan–Meier plot depicting disease-free survival according to neoadjuvant regimen.

When stratified by neoadjuvant regimen, no significant differences in DFS were observed (log-rank *p* = 0.416 for AC → TH vs. AC → THP, and *p* = 0.338 for AC → TH vs. TCHP) (Figure 2). During follow-up, no events were reported in the AC → TH (0/9) and TCHP (0/23) groups, while 5 events occurred in the AC → THP group (5/70). In the AC → THP group, the cumulative DFS rate at 42 months was 89.9% (SE = 0.047). In contrast, the survival curve for the TCHP group remained completely stable, with no progression events recorded.

The recurrence proportions were 7.1% (95% CI: 3.1–15.7) in the AC → THP group, <0% (95% CI: 0–14.3) in the TCHP group, and 0% (95% CI: 0–29.9) in the AC → TH group. These confidence intervals were wide and overlapping, reflecting the limited number of DFS events and the low statistical power of subgroup comparisons.

Overall, all three regimens were associated with high survival rates, with the longest event-free survival observed in the TCHP group. Still, the limited number of observed events constrains the statistical strength of subgroup analyses, and no significant differences were detected between treatment groups. It is noteworthy that all patients who experienced recurrence had tumors with a Ki-67 index ≥ 14%, although this association was not statistically significant.

## 4. Discussion

This multicenter study across nine oncology centers suggested that adjuvant T-DM1 is associated with favorable survival in individuals with early-stage HER2-positive breast cancer who had residual disease following neoadjuvant therapy. Survival outcomes appeared similar between patients treated with anthracycline-based and anthracycline-free regimens, indicating that the benefit of T-DM1 may extend across different therapeutic backbones in routine practice.

The CTNeoBC pooled analysis confirmed that achieving a total pCR is strongly prognostic in HER2-positive breast cancer [7]. In our study, breast and axillary pCR were assessed separately, and the observed rates were modest compared with prospective neoadjuvant trials. Nevertheless, survival outcomes remained favorable, supporting the concept that adjuvant escalation with T-DM1 can mitigate the poor prognosis of residual disease [8]. Interestingly, relapse was not encountered in patients with breast pCR, and was exceedingly rare among those with axillary pCR. Although these findings are based on very few events, they raise the possibility that the localization of residual disease may influence recurrence risk, an aspect that merits further validation in larger studies.

De-escalation trials such as PHARE and PERSEPHONE demonstrated that even patients with higher baseline risk continued to benefit from the standard duration of trastuzumab therapy [4]. In our cohort, a higher rate of recurrence was observed among patients who received fewer cycles of adjuvant T-DM1. This finding is noteworthy and should be confirmed in larger patient series. Similarly, the NeoSphere and TRYPHAENA trials showed that combining trastuzumab with pertuzumab markedly improved pCR rates [6,13]. The lower pCR rates observed in our cohort, compared with these prospective studies, most likely reflect the real-world inclusion of patients with more advanced clinical stages. Nevertheless, the favorable survival outcomes achieved suggest that adjuvant T-DM1 effectively compensates for this disadvantage, reinforcing its role in overcoming the poor prognosis typically associated with residual disease.

The pivotal KATHERINE trial established T-DM1 as the standard adjuvant option by halving recurrence risk compared with trastuzumab [8]. Subgroup analyses confirmed this benefit even among patients with HER2-negative residual disease [9], and pooled biomarker studies emphasized the prognostic significance of pCR and residual tumor [14]. Our study corroborates these results in daily practice, where patient populations are more heterogeneous and baseline stages are often more advanced.

An important observation was the absence of progression events in the small subgroup treated with TCHP, suggesting the potential effectiveness of this anthracycline-free regimen. This aligns with BCIRG-006, which demonstrated comparable efficacy but reduced cardiotoxicity when anthracyclines were omitted [15], and TRAIN-2, which confirmed non-inferior survival without anthracyclines [13,16]. TRYPHAENA also showed high pCR rates with TCHP [13]. Although subgroup numbers were limited, the favorable outcomes we observed with TCHP are consistent with prior reports supporting anthracycline-free backbones, especially when followed by T-DM1.

Our pCR rates were also lower than those observed in the WSG-ADAPT-HER2+/HR− trial, which reported substantially higher pathological response with early dual blockade [17]. Nevertheless, survival in our cohort remained excellent, again underscoring the ability of T-DM1 to compensate for residual disease.

Toxicity in our study was low, with only a small proportion experiencing severe adverse events. This mirrors results from ATEMPT [10] and MARIANNE [18], where T-DM1 was generally well tolerated, and also from TH3RESA, which showed safety even in heavily pretreated metastatic patients [19]. Notably, thrombocytopenia was uncommon in our cohort but has been linked to treatment persistence and outcomes in Turkish multicenter data [20]. In our real-world cohort, grade ≥ 3 adverse events were infrequent (<5%), which is consistent with other retrospective series reporting rates around 8–10% [19,20,21], but lower than the 25.7% observed in the prospective KATHERINE trial [8]. This difference likely reflects the retrospective nature of data collection, shorter treatment exposure, and the more flexible dose adjustments typical of routine clinical practice. The concordance between our findings and prior reports confirms that T-DM1 is both effective and feasible in the adjuvant setting.

Real-world series provide valuable context. Spanish and Asian cohorts demonstrated invasive DFS rates above 85% with adjuvant T-DM1 [21,22], while the Turkish Oncology Group reported survival outcomes consistent with ours [23]. Data from India confirmed feasibility in resource-constrained systems [22]. Our low recurrence rate is consistent with, and in some cases superior to, these reports, reflecting high adherence to adjuvant therapy, radiotherapy, and endocrine therapy in our study population.

Beyond clinical endpoints, emerging biomarkers may refine treatment strategies. The HER2DX assay has identified patients at higher risk despite pCR [24], while pooled analyses have shown that residual risk depends on nodal status [25]. In our cohort, many patients presented with cN1 disease, yet survival remained favorable, suggesting that T-DM1 can overcome risk factors identified by these models. Intratumor HER2 heterogeneity has also been associated with reduced pCR [26], which may partly explain our modest pathological response, while immune biomarkers such as tumor-infiltrating lymphocytes correlate with better outcomes [27]. The favorable DFS achieved in our cohort despite modest pCR may reflect these underlying biological mechanisms. In addition, although not statistically significant, all patients who relapsed in our cohort had tumors with Ki-67 ≥ 14%, suggesting that proliferative activity may continue to influence recurrence risk even in the context of adjuvant T-DM1.

The broader consistency of HER2-targeted therapy across disease settings strengthens confidence in our results. CLEOPATRA confirmed pertuzumab’s survival benefit in metastatic disease [28], providing a rationale for its inclusion in our neoadjuvant regimens. Historical adjuvant trials such as NSABP B-31 and BCIRG-006 established trastuzumab as transformative [15,29], and subsequent escalation trials like KATHERINE cemented T-DM1 as the standard [8,9,14]. Consensus recommendations from St. Gallen 2021 and major reviews highlight the importance of escalation with T-DM1 in high-risk patients [8,25,26]. Our findings, consistent with this body of evidence, show that even in real-world patients with advanced baseline stage, adjuvant T-DM1 delivers survival outcomes comparable to those reported in clinical trials.

This study has limitations, including its retrospective design, limited subgroup sizes, and heterogeneity of neoadjuvant regimens. As with all retrospective analyses, potential selection, information, and confounding biases due to unbalanced baseline characteristics between treatment groups cannot be excluded. Some clinicopathological variables were missing, and no imputation was applied, reflecting the inherent limitations of retrospective data collection. Small subgroup sizes reduced the power to detect between-group differences, and non-significant results should not be interpreted as evidence of equivalence. Furthermore, the relatively short follow-up may underestimate late recurrences, particularly in hormone receptor-positive tumors where relapse risk persists beyond the early years. Although the recurrence rate observed in our cohort was very low, longer follow-up is essential to confirm the durability of these favorable outcomes. Despite these constraints, the multicenter design and alignment with international evidence support the reliability of our conclusions. Given the retrospective and exploratory nature of this study, the results should be considered hypothesis-generating rather than confirmatory.

## 5. Conclusions

Adjuvant T-DM1 was associated with favorable survival outcomes in individuals with early-stage HER2-positive breast cancer who had residual disease following neoadjuvant treatment, across the different neoadjuvant regimens represented in our cohort. Anthracycline-free strategies such as TCHP yielded favorable outcomes in our cohort, supporting evidence from prospective and real-world studies. Toxicity was low and manageable, reinforcing feasibility in daily practice. Together with international trial data, biomarker insights, and consensus recommendations, our findings support the established role of T-DM1 as the standard of care while highlighting the need for confirmation in larger real-world series.

## Figures and Tables

**Table 1:** Baseline demographic, clinical, and pathological parameters of the cohort.

Variable		Mean ± SD (Min–Max)/*n* (%)
Age (years)		49.7 ± 11.0 (21.0–73.0)
Age category	<40	20 (19.6%)
	≥40	82 (80.4%)
BMI (kg/m^2^)		26.2 ± 5.0 (8.4–38.2)
ECOG performance status	0	79 (77.5%)
	1	23 (22.5%)
Comorbidity	Present	33 (32.4%)
	Absent	69 (67.6%)
Menopausal status	Postmenopausal	60 (58.8%)
	Premenopausal	42 (41.2%)
T stage	cT1	30 (29.4%)
	cT2	50 (49.0%)
	cT3	11 (10.8%)
	cT4	11 (10.8%)
N stage	cN0	23 (22.5%)
	cN1	47 (46.1%)
	cN2	27 (26.5%)
	cN3	5 (4.9%)
ER status	Unknown	2 (2.0%)
	Negative	18 (17.6%)
	Positive	82 (80.4%)
PR status	Unknown	2 (2.0%)
	Negative	24 (23.5%)
	Positive	76 (74.5%)
Ki-67 category	<14	16 (15.7%)
	≥14	86 (84.3%)
c-ErbB2	IHC 2+/FISH+	39 (38.2%)
	IHC 3+	63 (61.8%)

**Table 2:** Therapeutic approaches, surgical findings, and outcome indicators in the overall cohort.

Variable		Mean ± SD (Min–Max)/*n* (%)
Neoadjuvant regimen	AC + TH	9 (8.8%)
	AC + THP	70 (68.6)
	TCHP	23 (22.5%)
Histological grade	1	6 (5.9%)
	2	54 (52.9%)
	3	35 (34.3%)
	Unknown	7 (6.9%)
Histopathological type	Invasive ductal	99 (97.1%)
	Invasive lobular	2 (2.0%)
	Other (mixed)	1 (1.0%)
Lymphovascular invasion	Unknown	23 (22.5%)
	Present	37 (36.3%)
	Absent	42 (41.2%)
Perinodal invasion	Unknown	27 (26.5%)
	Present	29 (28.4%)
	Absent	46 (45.1%)
Multifocality	Present	22 (21.6%)
	Absent	80 (78.4%)
Type of surgery	BCS	47 (46.1%)
	MRM	55 (53.9%)
Breast pCR	Yes	6 (5.9%)
	No	96 (94.1%)
Axillary pCR	Yes	40 (39.2%)
	No	62 (60.8%)
Residual LN count *		5.0 ± 7.0 (0.0–36.0)
Dissected LN category	<10	65 (63.7%)
	≥10	37 (36.3%)
Neoadjuvant anthracycline	Yes	78 (76.5%)
	No	24 (23.5%)
Pertuzumab category	1–4 cycles	71 (69.6%)
	>4 cycles	22 (21.6%)
	None	9 (8.8%)
Adjuvant T-DM1 cycles	≤7	13 (12.9%)
	≥8	88 (87.1%)
Adjuvant radiotherapy	Yes	95 (93.1%)
	No	7 (6.9%)
Adjuvant endocrine therapy	Yes	81 (79.4%)
	No	21 (20.6%)
Follow-up (months)		33.9 ± 14.6 (13.0–67.0)
Recurrence/metastasis		5 (4.9%)

* Calculated only in patients without axillary pCR.

**Table 3:** Variation in clinical and pathological parameters across neoadjuvant regimens.

Variable	Category	AC + TH *n* (%)	AC + THP *n* (%)	TCHP *n* (%)	*p*-Value
Age category	<40	4 (44.4%)	13 (18.6%)	3 (13.0%)	0.139
	≥40	5 (55.6%)	57 (81.4%)	20 (87.0%)	
ECOG performance status	0	7 (77.8%)	53 (75.7%)	19 (82.6%)	0.863
	1	2 (22.2%)	17 (24.3%)	4 (17.4%)	
Comorbidity	Present	5 (55.6%)	24 (34.3%)	4 (17.4%)	0.096
	Absent	4 (44.4%)	46 (65.7%)	19 (82.6%)	
Menopausal status	Postmenopausal	5 (55.6%)	42 (60.0%)	13 (56.5%)	0.937
	Premenopausal	4 (44.4%)	28 (40.0%)	10 (43.5%)	
T stage	T1	7 (77.8%)	12 (17.1%)	11 (47.8%)	0.001
	T2	1 (11.1%)	39 (55.7%)	10 (43.5%)	
	T3	0 (0.0%)	11 (15.7%)	0 (0.0%)	
	T4	1 (11.1%)	8 (11.4%)	2 (8.7%)	
N stage	N0	7 (77.8%)	11 (15.7%)	5 (21.7%)	0.007
	N1	2 (22.2%)	33 (47.1%)	12 (52.2%)	
	N2	0 (0.0%)	23 (32.9%)	4 (17.4%)	
	N3	0 (0.0%)	3 (4.3%)	2 (8.7%)	
Tumor laterality	Right	7 (77.8%)	34 (48.6%)	13 (56.5%)	0.269
	Left	2 (22.2%)	36 (51.4%)	10 (43.5%)	
ER status	Unknown	0 (0.0%)	2 (2.9%)	0 (0.0%)	0.962
	Negative	2 (22.2%)	12 (17.1%)	4 (17.4%)	
	Positive	7 (77.8%)	56 (80.0%)	19 (82.6%)	
PR status	Unknown	0 (0.0%)	2 (2.9%)	0 (0.0%)	0.863
	Negative	2 (22.2%)	15 (21.4%)	7 (30.4%)	
	Positive	7 (77.8%)	53 (75.7%)	16 (69.6%)	
Ki-67 category	<14	1 (11.1%)	13 (18.6%)	2 (8.7%)	0.613
	≥14	8 (88.9%)	57 (81.4%)	21 (91.3%)	
c-ErbB2 IHC	2+	2 (22.2%)	31 (44.3%)	6 (26.1%)	0.174
	3+	7 (77.8%)	39 (55.7%)	17 (73.9%)	
Histological grade	Grade 1	2 (22.2%)	3 (4.3%)	1 (4.3%)	0.434
	Grade 2	4 (44.4%)	36 (51.4%)	14 (60.9%)	
	Grade 3	2 (22.2%)	26 (37.1%)	7 (30.4%)	
	Unknown	1 (11.1%)	5 (7.1%)	1 (4.3%)	
Histopathological type	Invasive ductal	9 (100.0%)	67 (95.7%)	23 (100.0%)	1.000
	Invasive lobular	0 (0.0%)	2 (2.9%)	0 (0.0%)	
	Other (mixed)	0 (0.0%)	1 (1.4%)	0 (0.0%)	
Lymphovascular invasion	Present	2 (22.2%)	29 (41.4%)	6 (26.1%)	0.604
	Absent	5 (55.6%)	26 (37.1%)	11 (47.8%)	
	Unknown	2 (22.2%)	15 (21.4%)	6 (26.1%)	
Perinodal invasion	Present	0 (0.0%)	24 (34.3%)	5 (21.7%)	0.137
	Absent	7 (77.8%)	27 (38.6%)	12 (52.2%)	
	Unknown	2 (22.2%)	19 (27.1%)	6 (26.1%)	
Multifocality	Present	2 (22.2%)	17 (24.3%)	3 (13.0%)	0.536
	Absent	7 (77.8%)	53 (75.7%)	20 (87.0%)	

**Table 4:** Distribution of treatment characteristics and pathological response patterns across neoadjuvant regimens.

Variable	Category	AC + TH *n* (%)	AC + THP *n* (%)	TCHP *n* (%)	*p*-Value
Type of surgery	BCS	7 (77.8%)	27 (38.6%)	13 (56.5%)	0.044
	MRM	2 (22.2%)	43 (61.4%)	10 (43.5%)	
Breast pCR	Yes	0 (0.0%)	3 (4.3%)	3 (13.0%)	0.205
	No	9 (100.0%)	67 (95.7%)	20 (87.0%)	
Axillary pCR	Yes	6 (66.7%)	27 (38.6%)	7 (30.4%)	0.465
	No	3 (33.3%)	43 (61.4%)	16 (69.6%)	
Dissected LN category	<10	7 (77.8%)	43 (61.4%)	15 (65.2%)	0.622
	≥10	2 (22.2%)	27 (38.6%)	8 (34.8%)	
Adjuvant T-DM1 category	≤7 cycles	1 (11.1%)	8 (11.4%)	4 (17.4%)	0.786
	≥8 cycles	8 (88.9%)	62 (88.6%)	18 (78.3%)	
Adjuvant radiotherapy	Yes	9 (100.0%)	65 (92.9%)	21 (91.3%)	1.000
	No	0 (0.0%)	5 (7.1%)	2 (8.7%)	
Adjuvant endocrine therapy	Yes	7 (77.8%)	56 (80.0%)	18 (78.3%)	1.000
	No	2 (22.2%)	14 (20.0%)	5 (21.7%)	
Recurrence/metastasis	Yes	0 (0.0%)	5 (7.1%)	0 (0.0%)	0.579
	No	9 (100%)	65 (92.9%)	23 (100%)	

**Table 5:** Range of T-DM1-related toxicities documented in the study group.

Variable		*n* (%)
Adverse events	None	21 (20.6%)
	Grade 1–2	77 (75.5%)
	Grade 3–4	4 (3.9%)
RT-related skin toxicity	None	91 (89.2%)
	Grade 1–2	10 (9.8%)
	Grade 3–4	1 (1.0%)
Thrombocytopenia	None	73 (71.6%)
	Grade 1–2	24 (23.5%)
	Grade 3–4	5 (4.9%)
Anemia	None	76 (74.5%)
	Grade 1–2	23 (22.5%)
	Grade 3–4	3 (2.9%)
Leukopenia	None	82 (80.4%)
	Grade 1–2	17 (16.7%)
	Grade 3–4	3 (3.0%)
Elevated liver enzymes	None	66 (64.7%)
	Grade 1–2	31 (30.4%)
	Grade 3–4	5 (4.9%)
Hypokalemia	None	97 (95.1%)
	Grade 1–2	5 (4.9%)
	Grade 3–4	0 (0.0%)

**Table 6:** Distribution of T-DM1–related adverse events according to prior neoadjuvant therapy.

Adverse Events	Category	AC + TH *n* (%)	AC + THP *n* (%)	TCHP *n* (%)	*p*-Value
Overall adverse events	None	2 (22.2%)	14 (20.0%)	5 (21.7%)	0.018
	Grade 1–2	5 (55.6%)	56 (80.0%)	16 (69.6%)	
	Grade 3–4	2 (22.2%)	0 (0.0%)	2 (8.7%)	
Nausea/vomiting	None	8 (88.9%)	48 (68.6%)	17 (73.9%)	0.782
	Grade 1–2	1 (11.1%)	18 (25.7%)	6 (26.1%)	
	Grade 3–4	0 (0.0%)	4 (5.7%)	0 (0.0%)	
Headache	None	9 (100.0%)	63 (90.0%)	20 (87.0%)	0.760
	Grade 1–2	0 (0.0%)	7 (10.0%)	3 (13.0%)	
	Grade 3–4	0 (0.0%)	0 (0.0%)	0 (0.0%)	
Myalgia	None	7 (77.8%)	50 (71.4%)	15 (65.2%)	0.305
	Grade 1–2	1 (11.1%)	19 (27.1%)	8 (34.8%)	
	Grade 3–4	1 (11.1%)	1 (1.4%)	0 (0.0%)	
Hypertension	None	7 (77.8%)	66 (94.3%)	20 (87.0%)	0.189
	Grade 1–2	2 (22.2%)	3 (4.3%)	2 (8.7%)	
	Grade 3–4	0 (0.0%)	1 (1.4%)	1 (4.3%)	
RT-related skin toxicity	None	8 (88.9%)	65 (92.9%)	18 (78.3%)	0.157
	Grade 1–2	1 (11.1%)	4 (5.7%)	5 (21.7%)	
	Grade 3–4	0 (0.0%)	1 (1.4%)	0 (0.0%)	
Thrombocytopenia	None	4 (44.4%)	54 (77.1%)	15 (65.2%)	0.167
	Grade 1–2	4 (44.4%)	13 (18.6%)	7 (30.4%)	
	Grade 3–4	1 (11.1%)	3 (4.3%)	1 (4.3%)	
Anemia	None	8 (88.9%)	48 (68.6%)	20 (87.0%)	0.218
	Grade 1–2	1 (11.1%)	20 (28.6%)	2 (8.7%)	
	Grade 3–4	0 (0.0%)	2 (2.9%)	1 (4.3%)	
Leukopenia	None	7 (77.8%)	58 (82.9%)	17 (73.9%)	0.351
	Grade 1–2	1 (11.1%)	11 (15.7%)	5 (21.7%)	
	Grade 3–4	1 (11.1%)	1 (1.4%)	1 (4.3%)	
Elevated liver enzymes	None	5 (55.6%)	48 (68.6%)	13 (56.5%)	0.077
	Grade 1–2	2 (22.2%)	21 (30.0%)	8 (34.8%)	
	Grade 3–4	2 (22.2%)	1 (1.4%)	1 (8.6%)	
Hypokalemia	None	9 (100%)	66 (94.3%)	22 (95.7%)	1.000
	Grade 1–2	0 (0.0%)	4 (5.7%)	1 (4.3%)	
	Grade 3–4	0 (0.0%)	0 (0.0%)	0 (0.0%)	

## Data Availability

The data that support the findings of this study are not openly available due to reasons of sensitivity and patient privacy, but are available from the corresponding author upon reasonable request.

## Supplementary Materials

The following supporting information can be downloaded at: https://www.mdpi.com/article/10.3390/medicina61111966/s1, Table S1: Baseline laboratory parameters before adjuvant T-DM1.

## Abbreviations

The following abbreviations are used in this manuscript:

AC	Doxorubicin (Adriamycin) + Cyclophosphamide
BCS	Breast-Conserving Surgery
BMI	Body Mass Index
ECOG	Eastern Cooperative Oncology Group
ER	Estrogen Receptor
MAX	Maximum
MIN	Minimum
MRM	Modified Radical Mastectomy
PR	Progesterone Receptor
SD	Standart Deviation
