# Supplementary material for: Neoadjuvant Regimens and Their Impact on Adjuvant T-DM1 Outcomes in HER2-Positive Early Breast Cancer
Source: Medicina (Kaunas). 2025 Nov 1;61(11):1966. doi: 10.3390/medicina61111966 (PMC12654407; doi:10.3390/medicina61111966)
Supplement: Supplementary file 1 [file medicina-61-01966-s001.zip › medicina-3920818-supplementary.pdf]

**Table S1.** Baseline laboratory parameters before adjuvant T-DM1

| **Varıable** | **Mean ± SD (Min-Max)** |
|---|---|
| Neutrophils (/µL) | 4245.8 ± 1651.0 (1220–9910) |
| Lymphocytes (/µL) | 1913.1 ± 794.6 (400–5740) |
| Platelets (×$10^9$/L) | 258.5 ± 79.5 (89–530) |
| Eosinophils (/µL) | 159.8 ± 123.5 (0–600) |
| Monocytes (/µL) | 473.2 ± 189.5 (0–1230) |
| MPV (fL) | 10.6 ± 9.2 (7.3–102) |
| Hemoglobin (g/dL) | 12.3 ± 1.3 (8.5–15.5) |
| Albumin (g/dL) | 4.7 ± 4.2 (3.5–47) |
| Globulin (g/dL) | 3.1 ± 0.4 (2.0–4) |
| CRP (mg/dL) | 3.2 ± 3.6 (0.1–22.9) |
| ALP (U/L) | 81.8 ± 23.4 (37–164) |
| LDH (U/L) | 195.9 ± 44.8 (118.0–359) |
| ALT (U/L) | 23.4 ± 10.5 (6.0–71) |
| Total bilirubin (mg/dL) | 0.5 ± 0.3 (0.1–1.1) |
| Creatinine (mg/dL) | 0.7 ± 0.3 (0.3–2.9) |
| GFR (mL/min/1.73$m^2$) | 99.3 ± 17.6 (13–132) |
| CEA (ng/mL) | 3.1 ± 2.4 (0.4–17.8) |
| CA15-3 (U/mL) | 16.3 ± 7.3 (5.3–53.5) |
